# The E2F activators control multiple mitotic regulators and maintain genomic integrity through Sgo1 and BubR1

**DOI:** 10.18632/oncotarget.20765

**Published:** 2017-09-08

**Authors:** Miyoung Lee, Yainyrette Rivera-Rivera, Carlos S. Moreno, Harold I. Saavedra

**Affiliations:** ^1^ Aflac Cancer and Blood Disorders Center, Department of Pediatrics, Emory University School of Medicine, Atlanta, Georgia 30322, USA; ^2^ Department of Pathology and Laboratory Medicine, Emory University School of Medicine, Atlanta, Georgia 30322, USA; ^3^ Department of Basic Sciences, Program of Pharmacology, Ponce Health Sciences University-School of Medicine/Ponce Research Institute, Ponce, 00716-2348 Puerto Rico

**Keywords:** centrosome, transcription factors, chromosome instability, breast cancer, mammary epithelial cells

## Abstract

The E2F1, E2F2, and E2F3a transcriptional activators control proliferation. However, how the E2F activators regulate mitosis to maintain genomic integrity is unclear. Centrosome amplification (CA) and unregulated spindle assembly checkpoint (SAC) are major generators of aneuploidy and chromosome instability (CIN) in cancer. Previously, we showed that overexpression of single E2F activators induced CA and CIN in mammary epithelial cells, and here we show that combined overexpression of E2F activators did not enhance CA. Instead, the E2F activators elevated expression of multiple mitotic regulators, including Sgo1, Nek2, Hec1, BubR1, and Mps1/TTK. cBioPortal analyses of the TCGA database showed that E2F overexpression in lobular invasive breast tumors correlates with overexpression of multiple regulators of chromosome segregation, centrosome homeostasis, and the SAC. Kaplan-Meier plots identified correlations between individual or combined overexpression of E2F1, E2F3a, Mps1/TTK, Nek2, BubR1, or Hec1 and poor overall and relapse-free survival of breast cancer patients. In MCF10A normal mammary epithelial cells co-overexpressing E2Fs, transient Sgo1 knockdown induced CA, high percentages of premature sister chromatid separation, chromosome losses, increased apoptosis, and decreased cell clonogenicity. BubR1 silencing resulted in chromosome losses without CA, demonstrating that Sgo1 and BubR1 maintain genomic integrity through two distinct mechanisms. Our results suggest that deregulated activation of the E2Fs in mammary epithelial cells is counteracted by activation of a Sgo1-dependent mitotic checkpoint.

## INTRODUCTION

Regulation of mitotic function is central to cancer control, and tumors often display increased expression of mitotic regulators [1, 2]. Currently, small-molecule inhibitors against centrosome and mitotic regulators are in clinical trials, with inhibitors against Plk1 and Aurora kinase A being particularly effective [3, 4]. Others, including inhibitors against Mps1/TTK or Nek2, have been proven effective in mouse models of cancer [5–11]. Multiple proteins regulate mitosis [12–20]. For example, the centrosome duplication cycle, which is regulated by a plethora of transcription factors, cyclin-dependent kinases, and centrosome-specific kinases and phosphatases, results in two centrosomes that promote the formation of a bipolar mitotic spindle and equal segregation of chromosomes [21–25]. In addition, cyclin B/Cdk1 triggers entry to mitosis and its inactivation is required for mitotic exit [20]. Proteins involved in the spindle assembly checkpoint (SAC), which monitors misaligned chromosomes at metaphase, include Aurora kinase B (AURKB), Mps1/TTK, NDC80 (Hec-1, a phosphorylation target of Nek2), KNL1, BubR1 (or BUB1B), Bub3, Bub1, Mad1, Mad2, and Cdc20 [26]. Additionally, PP2A and BubR1 co-localize with Sgo1 to centromeres [17, 27], where Sgo1 protects chromosome cohesion by protecting cohesin from premature detachment from centromeres [28, 29]. Sgo1 is also phosphorylated by Nek2, an event also necessary for chromosome alignment, and by AURKB, which allows shuttling of Sgo1 between chromosome arms and centromeres [15, 30, 31].

Breast cancers and breast cancer cell lines overexpress several mitotic regulators, including kinases that regulate the SAC such as Nek2, Mad1L1, Mad2L1, Mad2L2, BubR1, BubR1B, Bub3, Cdc20, and Mps1/TTK [32–34]. Consistent with unregulated mitotic proteins as cancer drivers, overexpression of Aurora A in mammary epithelial cells of rodents causes mammary tumors [35, 36], overexpression of NDC80 triggers lung and hepatocellular adenomas and sarcomas [37], and overexpression of PTTG1 (securin) induces hyperplasia and microadenomas of the pituitary [38]. Likewise, overexpression of Mad2 accelerates lung tumorigenesis initiated by the *K-Ras* oncogene [39] and lymphomas induced by c-Myc [40].

Because deregulated mitotic kinases may play key roles in breast cancer, it is important to find mechanisms driving their deregulation. The activities and expression of the E2F transcriptional activators E2F1, E2F2, and E2F3a reach maximal levels at late G1 and S phases and regulate gene expression of proteins involved in cell cycle progression, differentiation, DNA repair, cell survival, and the centrosome cycle [41–45]. Because they control the cellular processes listed above, the Rb-E2F pathway is frequently deregulated in human tumors, and multiple mouse models have demonstrated that overexpression of E2Fs initiates and maintains tumors originating from distinct tissues [41, 46–50]. Although E2F overexpression is generally thought to be tumor promoting, in some tissue types such as the skin they are tumor suppressive, which is tightly linked to the induction of apoptosis in that particular tissue [51]. The E2F activators were initially characterized by their ability to drive quiescent cells into S phase [52–55]; however, how they regulate mitosis is less understood. The first clue of E2F activator involvement in mitosis was derived from microarray analyses, which identified multiple drivers of DNA proliferation and a smaller number of genes that regulate mitosis [56–58]. Other clues were that the E2F1 activator and the E2F4 repressor bind to the promoters of G1, S, G2, and M phase regulators, and both transcription factors bind the *Cdk1* promoter region [57]. Furthermore, level of cyclin B1 is controlled by E2F1 and cyclin A through rearrangement of the anaphase-promoting complex (APC), whereas APC controls E2F1 degradation in prometaphase [59, 60].

Despite evidence showing that E2F activators regulate the expression of genes controlling mitosis, functional evidence is minimal and mechanisms are unknown. For example, silencing E2F3 prevented entry into G2/M in ovarian cancer cells [61]. Our laboratory showed that silencing E2F3 in HCC1954 Her2^+^ breast cancer cells resulted in a significant delay in the completion of cytokinesis [62] and that tumor suppression triggered by silencing E2F3 in breast cancer cells is strongly associated with significant reductions in percentages of mitotic cells [63]. We propose that at least two major mechanisms may contribute to the deregulation of mitosis and chromosome instability (CIN) by the E2F activators: the E2Fs directly affect the expression of proteins that regulate the mitotic machinery or indirectly affect mitotic progression through inducing centrosome amplification (CA), an abnormal cellular process in which cells acquire three or more centrosomes [22]. CA results in multipolar mitosis, which consequences may include mitotic catastrophe or delayed mitotic progression [64, 65]. Aberrant mitoses may also result in the acquisition of aneuploidy and CIN [66, 67]. Our laboratory has demonstrated that deregulation of regulators of the centrosome cycle, mitosis, and G1/S phase including Cdk4, the E2F activators (E2F1, E2F3a), Nek2, Sgo1, and Mps1/TTK are required to maintain high CA and CIN in Her2^+^ breast cancer cells [34, 62, 68].

In this study, by searching for suppressors of CA and CIN in mammary epithelial cells expressing all E2F activators, we found that the E2F activators control the expression of multiple mitotic regulators. Silencing Sgo1 in mammary epithelial cells overexpressing E2F1, E2F2, and E2F3a resulted in chromosome missegregation and CA, thereby suggesting a role for Sgo1 in preventing CA triggered by the E2Fs. On the other hand, silencing of BubR1 resulted in chromosome missegregation without triggering CA. Our results suggest that BubR1 and Sgo1 maintain genomic integrity downstream of the E2F activators through different mechanisms.

## RESULTS

### Combined E2F overexpression does not enhance centrosome amplification in mammary epithelial cells

**Figure 1:**
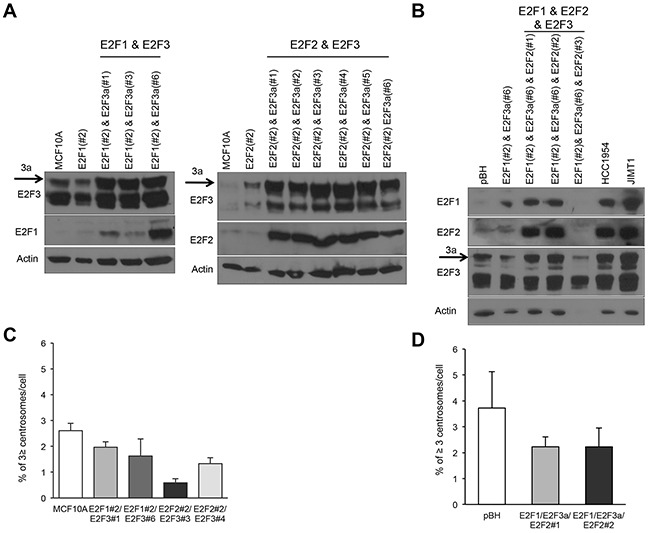
Combined overexpression of the transcriptional activators E2F1, E2F2, and E2F3a does not induce centrosome amplification in mammary epithelial cells **(A)** Cells overexpressing two E2F activators were generated by transfecting E2F3a (pBABE-puro-E2F3a) into cells overexpressing E2F1 or E2F2 [62]. **(B)** Cells overexpressing E2F1, E2F2, and E2F3a were generated by transfecting E2F2 (pcDNA3.1/3x myc-A-E2F2) into cells overexpressing E2F1 and E2F3a. **(C, D)** The centrosome amplification assay detects percentages of cells displaying ≥3 centrosomes and was performed by immunostaining cells with pericentrin, counterstaining nuclei with DAPI, and counting 200 cells in three independent experiments. (# represents the specific population of each cell line and numbers are given to indicate that populations are independent).

MCF10A is a non-transformed mammary cell line that displays a functional p53 pathway, has low frequencies of CA and CIN [33, 34, 62, 69, 70] and differentiates into normal acinar structures in 3D cultures [71]. In a previous study, we demonstrated that E2F1, E2F2, and E2F3a are highly deregulated in breast cancer and their individual overexpression induced CA and CIN in MCF10A mammary epithelial cells [68]. To identify the functional consequences of co-upregulation of E2F1, E2F2, and E2F3 in breast tumors—specifically to identify drivers of CA and mitotic dysfunction in mammary epithelial cells—we engineered MCF10A mammary epithelial cells to overexpress E2F1 and E2F3a, E2F2 and E2F3a, or the three E2F activators (Figure 1A and 1B). Levels of E2Fs in cells overexpressing combinations of E2Fs are more robust than in control cells or in cells overexpressing individual E2Fs. Levels of E2Fs in cells overexpressing combinations of E2Fs were similar to those expressed in the Her2^+^ breast cancer cell lines HCC1954 and JIMT-1 (Figure 1B), suggesting we have developed a system that mimics levels of E2Fs in breast cancer cells. Surprisingly, and in contrast to MCF10A cells overexpressing individual E2Fs [68], combined expression of E2Fs did not enhance CA (Figure 1C and 1D). The absence of CA in cells co-overexpressing E2Fs suggests the activation of a checkpoint that may actively suppress CA.

### The E2Fs regulate proteins involved in centrosome homeostasis, the spindle assembly checkpoint (SAC), and chromosome cohesion

**Figure 2:**
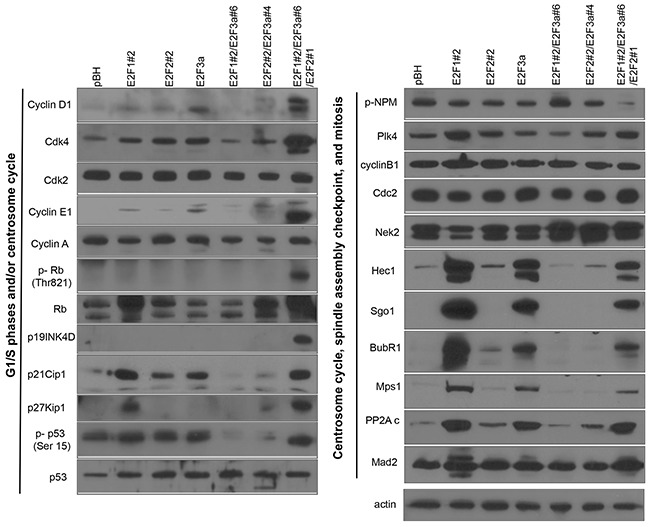
The E2Fs regulate spindle assembly checkpoint (SAC) and cell cycle and centrosome cycle regulators Cell protein lysates were prepared from actively proliferating cells, and 15 μg of protein was used to analyze multiple centrosome cycle, cell cycle, and SAC regulators.

To address how individual and combined expression of E2Fs affect gene expression in mammary epithelial cells with the objective of finding suppressors of CA and CIN, we performed Western blot analyses of proteins that regulate the centrosome cycle, G1/S phase, the SAC, and mitosis [14, 22, 23, 64] (Figure 2). Individual expression of E2Fs resulted in small changes in the expression of Cdk4, Plk4, cyclin D1, cyclin E, and p53 relative to cells expressing vector control. Co-expression of E2F1, E2F2, and E2F3 resulted in an outcome different from the expression of one or two E2F activators, since levels of p19^INK4D^, cyclin D1, Cdk4, cyclin E, Rb, and p-Rb-Thr-821, a substrate of Cdk4 [72], were higher than in cells expressing single E2Fs. Although upregulation of G1/S proteins by the E2Fs was expected, the most striking results from these analyses were the ability of single and/or combined overexpression of E2F activators to enhance the protein levels of multiple regulators of mitosis, including Nek2, NDC80 (Hec1), Sgo1, BubR1, and Mps1/TTK. Levels were similar between cells expressing single, or combined E2Fs, except for Nek2, which expression is higher in cells overexpressing the combinations of two or three E2Fs. In addition, the only cyclin-dependent kinase inhibitor (CKI) specifically activated by combined expression of the three E2F activators was p19^INK4D^.

### E2Fs are overexpressed in basal and Her2+ breast tumors and upregulation of E2Fs and their mitotic targets is associated with poor survival of breast cancer patients

**Figure 3:**
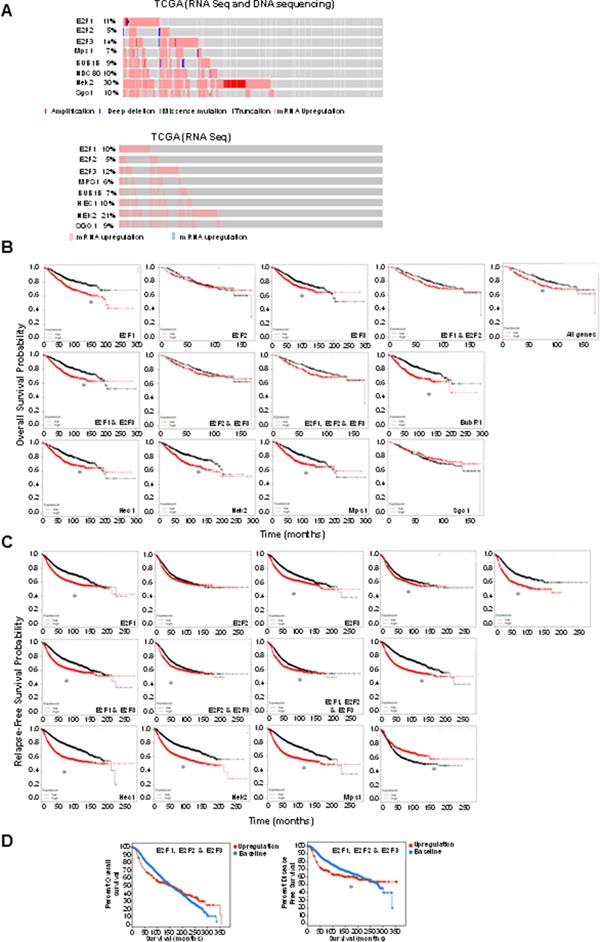
Overexpression of E2Fs and regulators of mitosis associate with poor survival of breast cancer patients **(A)** Oncoprint analysis of the 8 indicated genes using the cBioPortal program mining the TCGA database. Top panel: The analysis indicates the percentage of total alterations in 971 cases and the specific alteration (amplification, deep deletion, missense mutation, truncating mutation, and mRNA upregulation). Bottom panel: overexpression of mRNAs as determined by RNA seq. Kaplan-Meier graphs generated with KM Plotter displaying the relation between overexpression of the indicated proteins (red) and probability of overall **(B)** or relapse-free **(C)** survival relative to patients that do not overexpress the indicated proteins (black). Significance (log-rank P) for (B) is as follows: E2F1 was P = 1.6e-07, E2F2 was P = 0.92, E2F3 was P = 0.0036, E2F1 and E2F2 was P = 0.19, E2F1 and E2F3 was P = 5.1e-16, E2F2 and E2F3 was P = 0.47, E2F1 & E2F2 & E2F3 was P = 0.12, BubR1 was P = 1.6e-08, Hec1 was P = 4.8e-05, Nek2 was P = 1.3e-06, Mps1 was P = 4.8e-05, SgoI was P = 0. 2, and P = 0.036 for all genes. Log-rank P for (C) is as follows: E2F1 was P = 3.6e-13, E2F2 was P = 0.12, E2F3 was P = 8.9e-10, E2F1 and E2F2 was P = 0.042, E2F1 and E2F3 was P = 1e-13, E2F2 and E2F3 was P = 0.017, E2F1 and E2F2 and E2F3 was P = 0.0082, BubR1 was P < 1e-16, Hec1 was P < 1e-16, Nek2 was P < 1e-16, Mps1 was P < 1e-16, Sgo1 was P < 1.3e-05, and P < 5.2e-11 for all genes. **(D)** Kaplan-Meier curves generated in cBioPortal based on the METABRIC database displaying percentage of patient survival (y axis) indicated by months (x axis) that overexpress (red) E2F1, E2F2, and E2F3 versus patients who do not overexpress any of the genes (blue). P value for percent overall survival = 0.949, P value for relapse-free survival = 0.0250.

To address whether our observations in mammary epithelial cells are translated into breast cancers, we performed TCGA analysis on selected SAC proteins, including BubR1, NDC80, Sgo1, and Mps1/TTK in relation to E2Fs genes using cBioPortal [73, 74]. The TCGA database encompasses 971 lobular invasive breast cancer samples from the 2015 *Cell* publication [75]. Because the exact percentage of lobular invasive breast tumors displaying alterations in the E2Fs or mitotic proteins are unknown, we queried the database and found that E2F1 is altered in 11% of cases, E2F2 in 5%, E2F3 in 14%, Mps1/TTK in 7%, BubR1B in 9%, NDC80 in 10%, Nek2 in 30%, and Sgo1 in 10% of cases. Although a few cases with amplification (in particular Nek2 and E2F1), missense mutations, or deep deletions were identified (Figure 3A, top), the most common alterations were overexpression (Figure 3A, bottom). Figure 3A also indicates that there is significant overlap between E2F overexpression and overexpression of mitotic regulators. We next addressed whether at least two E2F activators were upregulated in a particular breast cancer subtype by mining the METABRIC database, which classified breast tumors by PAM50 intrinsic subtype analysis and found that higher percentages of basal, Her2+, and luminal B breast tumors co-overexpress at least two E2F activators (Table 1). Strikingly, 42% of basal and 18.3% of Her2+ breast cancers overexpress at least two E2Fs. This is highly significant, since basal and Her2+ are the breast cancer subtypes with the worst prognosis [76].

**Table 1:** Percentage intrinsic breast cancer subtypes that overexpress at least two E2F activators

Subtype	Count	% of all breast cancers (out of 1992 tumors)	% of intrinsic subtype
Basal	138	6.9%	42% (out of 331 basal)
Her2^+^	44	2.2%	18.3% (out of 240 Her2+)
Lum A	6	0.3%	0.83% (out of 721 luminal A)
Lum B	30	1.5%	6.1% (out of 492 luminal B)
Not classified	6	0.3%	100% (out of 6 not classified)
Normal	1	0.5%	0.5% (out of 202 normal)

Using cBioPortal analysis of the TCGA database, we next addressed whether individual genes co-occurred in breast cancers and found significant correlations between E2F1 overexpression and E2F2, E2F1 overexpression and E2F3, and E2F2 overexpression and E2F3. Also, E2F1, E2F2, E2F3, Mps1/TTK, BubR1, NDC80 (HEC1), Nek2, and Sgo1 significantly co-overexpress in breast tumors (Table 2). We used cBioPortal network analysis to address if co-overexpression of E2Fs and other mitotic proteins (Mps1/TTK, BubR1, NDC80 (HEC1), Nek2, and Sgo1) correlated with the overexpression of other cell cycle regulators. This analysis indicated that these proteins form a network with 276 other proteins. Table 3 illustrates a network that includes the eight queried genes (E2F1, E2F2, E2F3, BUBR1, Mps1/TTK, NDC80, Nek2, Sgo1) and the 50 most frequently altered neighbor genes as well as frequencies of each type of alteration. The network includes proteins involved in microtubule and mitotic spindle dynamics (AURKA, CSNK1D, MAPRE1, NDE1), proteins that induce CA when under/overexpressed (Brca1, RB1, AURKA, CP110, SKP2, TP53), centrosome regulators and structural proteins (CP110, AURKA, CEP250, MAPRE1, SDCCAG8, TUBGCP3), kinetochore and centromere proteins (CENPF, CENPL, DSN1, NUF2, NUP133, SKA2), transcription factors and transcriptional co-activators (AHCTF1, FOXM1, NCOA3, TFDP1, TP53), and G1/S phase regulators (CDC6, SKP2, p53, Rb, p107, Myc, FOXM1, RPS6KB1, PPP2R5A, PPP2R5D), among others.

**Table 2:** Co-occurrences between the indicated genes (C-BIOPORTAL/TCGA Analysis)

Gene A	Gene B	P Value (Fisher exact test)	Log Odds Ratio
**E2F1**	E2F2	<0.001	1.778
	E2F3	<0.001	1.342
	TTK	<0.001	2.090
	BUBR1	<0.001	1.620
	NDC80 (Hec1)	<0.001	2.028
	NEK2	<0.001	1.417
	SGO1	<0.001	1.969
**E2F2**	E2F3	<0.001	2.790
	TTK	<0.001	2.903
	BUBR1	<0.001	2.362
	NDC80 (Hec1)	<0.001	2.607
	NEK2	<0.001	1.970
	SGO1	<0.001	>3
**E2F3**	TTK	<0.001	2.952
	BUBR1	<0.001	2.019
	NDC80 (Hec1)	<0.001	2.411
	NEK2	<0.001	1.012
	SGO1	<0.001	1.826
**Mps1/TTK**	BUBR1	<0.001	2.567
	NDC80 (Hec1)	<0.001	2.714
	NEK2	<0.001	2.149
	SGO1	<0.001	2.861
**BUBR1B**	NDC80 (Hec1)	<0.001	2.726
	NEK2	<0.001	1.797
	SGO1	<0.001	2.463
**NDC80**	NEK2	<0.001	1.448
	SGO1	<0.001	2.318
**NEK2**	SGO1	<0.001	2.233

**Table 3:** Network that includes the 8 query genes (E2F1, E2F2, E2F3, BUBR1, TTK, NDC80, Nek2, Sgo1) and the 50 most frequently altered neighbor genes (out of 279)

Gene	Function	Total Alterations	Amplification	Homozygous Deletion	Up-regulation	Down-regulation	Mutation
**AHCTF1**	Putative AT-hook-containing transcription factor	36.5%	14.6%	0.1%	29.6%	0.4%	1.6%
**AURKA**	Microtubule formation and/or stabilization, centrosome homeostasis	20.4%	5.6%	0.0%	18.6%	0.0%	0.5%
**BIRC5**	Inhibitor of apoptosis (IAP) gene family	15.3%	6.2%	0.1%	12.9%	0.0%	0.0%
**BRCA1**	DNA repair, centrosome amplification, chromosome instability	13.5%	1.8%	0.8%	9.7%	0.0%	2.5%
**BUBR1**	Spindle checkpoint	8.7%	0.4%	1.1%	7.0%	0.0%	0.3%
**CP110**	Centriolar protein	16.5%	4.3%	0.0%	13.6%	0.0%	0.4%
**CDC16**	Ubiquitin ligase, component of the APC complex	24.6%	2.5%	0.7%	9.1%	14.9%	0.3%
**CDC6**	Early DNA replication	15.3%	6.5%	0.3%	13.3%	0.0%	0.3%
**CENPF**	Centromere-kinetochore, spindle, midzone	25.5%	12.5%	0.0%	15.6%	0.0%	1.3%
**CENPL**	Kinetochore, mitotic progression	28.1%	10.0%	0.0%	22.0%	0.0%	0.5%
**CEP250**	Centriole-centriole cohesion	18.4%	2.8%	0.1%	15.0%	1.3%	1.2%
**CREBBP**	Histone acetyl-transferase, transcriptional co-activation	20.8%	5.1%	0.4%	14.0%	1.9%	2.0%
**CSNK1D**	DNA replication and repair, apoptosis, microtubule dynamics, chromosome segregation	16.8%	6.2%	0.6%	10.1%	3.4%	0.1%
**DSN1**	Kinetochore assembly and progression through the cell cycle	15.9%	2.0%	0.0%	15.0%	0.0%	0.4%
**E2F1**	Cell cycle, centrosome amplification	10.6%	1.2%	0.1%	9.3%	0.0%	0.6%
**E2F2**	Cell cycle, centrosome amplification	4.9%	0.0%	0.4%	4.5%	0.0%	0.0%
**E2F3**	Cell cycle, centrosome amplification	13.7%	2.3%	0.2%	12.8%	0.0%	0.2%
**FOXM1**	Cell proliferation	15.2%	2.8%	0.1%	14.5%	0.0%	0.6%
**KLHL12**	May act as a substrate adaptor of the Cullin-3 ubiquitin ligase complex	35.2%	12.9%	0.0%	29.7%	0.4%	0.3%
**LRRC59**	Ribosome binding protein	20.2%	7.8%	0.1%	19.4%	0.0%	0.1%
**MAPRE1**	Microtubules, dynactin complex, mitotic centrosomes and spindle microtubules	19.1%	1.5%	0.1%	18.3%	0.4%	0.1%
**MCL1**	Anti-apoptotic protein	19.3%	14.4%	0.0%	9.7%	0.0%	0.1%
**MDM4**	Binds and inhibits p53, suppresses MDM2 function	26.4%	13.9%	0.0%	18.3%	0.0%	0.3%
**MYC**	Cell cycle progression, apoptosis	26.2%	21.9%	0.0%	6.7%	0.0%	0.3%
**NCOA3**	Histone acetyltransferase, transcriptional co-activator	21.2%	3.8%	0.0%	16.4%	0.3%	4.7%
**NCOA6**	Transcriptional co-activator	17.9%	2.0%	0.0%	14.7%	1.5%	1.8%
**NDC80 (Hec-1)**	Chromosome segregation	10.3%	10.3%	0.5%	9.5%	0.0%	0.4%
**NDE1**	Microtubule organization, mitosis and neuronal migration	14.7%	4.5%	0.0%	11.4%	0.2%	0.2%
**NEK2**	Centrosome separation, mitotic checkpoint	29.6%	12.3%	0.0%	21.7%	0.0%	0.1%
**NSL1**	Kinetochores	39.4%	12.0%	0.0%	34.6%	1.0%	0.5%
**NUF2**	Centromere	26.6%	12.4%	0.0%	19.8%	0.0%	0.3%
**NUP107**	Nuclear pore complex	13.3%	3.3%	0.0%	11.3%	0.4%	0.9%
**NUP133**	Nuclear envelope, kinetochores	38.9%	13.7%	0.0%	33.6%	1.0%	0.3%
**NUP85**	Nuclear pore complex	19.6%	6.2%	0.0%	17.7%	0.1%	0.1%
**PARP1**	DNA damage response	37.3%	13.6%	0.0%	29.9%	0.1%	0.9%
**PHB**	Cellular senescence	16.4%	8.1%	0.1%	14.5%	0.0%	
**PMF1**		25.5%	11.3%	0.0%	18.1%	0.0%	0.0%
**PPP2CB**	Phosphatase, negative control of cell cycle	24.0%	1.8%	4.7%	5.6%	15.9%	0.3%
**PPP2R5A**	Phosphatase, negative control of cell cycle	22.6%	12.0%	0.0%	14.7%	0.0%	0.1%
**PPP2R5D**	Phosphatase involved in negative control of cell cycle	17.8%	2.1%	0.3%	15.1%	2.2%	0.4%
**PRKDC**	DNA double strand break repair and recombination	24.1%	6.8%	0.1%	18.6%	0.0%	2.1%
**RB1**	Regulator of the cell cycle, centrosome amplification	14.7%	0.2%	4.5%	2.9%	7.6%	2.3%
**RBL1 (p107)**	Negative regulator of the cell cycle	13.9%	2.1%	0.1%	12.4%	0.0%	0.8%
**RPL7**	Ribosomal protein	23.2%	10.4%	0.1%	18.1%	0.0%	0.1%
**RPS27**	Ribosomal protein	19.2%	12.2%	0.0%	8.9%	0.0%	0.0%
**RPS6KB1**	Protein synthesis, cell growth, and cell proliferation	25.0%	11.0%	0.0%	22.5%	1.2%	0.4%
**SDCCAG8**	Organizing the centrosome	33.6%	14.5%	0.0%	24.9%	1.2%	0.7%
**SGO1**	Protects centromeric cohesin from cleavage	10.3%	1.2%	0.1%	9.7%	0.0%	0.2%
**SKA2**	Spindle and kinetochore	21.2%	9.0%	0.1%	18.7%	0.0%	0.0%
**SKP2**	Ubiquitination, degradation of p27^KIP1^	13.2%	2.0%	0.3%	11.9%	0.0%	0.2%
**TFDP1**	Binds E2Fs and enhances their DNA binding	13.5%	2.6%	0.5%	11.2%	1.1%	0.5%
**TK1**	Thymidine kinase	14.4%	6.2%	0.1%	11.3%	0.0%	0.1%
**TOM1L1**	Src activating and signaling molecule	14.4%	7.2%	0.0%	12.3%	0.4%	0.3%
**TP53**	Cell cycle arrest, apoptosis, senescence, DNA repair, centrosome amplification	38.0%	0.1%	1.3%	5.0%	3.4%	35.0%
**Mps1/TTK**	Centrosome duplication, spindle assembly checkpoint	7.4%	0.2%	0.2%	6.8%	0.0%	0.4%
**TUBGCP3**	Tubulin gamma complex-associated protein	16.0%	2.6%	0.4%	10.0%	4.8%	0.5%
**UBE2C**	E2 ubiquitin-conjugating enzyme family, cell cycle progression	13.2%	3.2%	0.1%	11.2%	0.0%	0.0%
**XRCC6**	Repair of non-homologous DNA ends	16.8%	0.3%	0.1%	7.1%	9.1%	0.6%

Next we queried *Kaplan-Meier* plotter (a database that encompasses gene expression and survival data of genes in 4,142 breast cancer patients where data have been downloaded from GEO, EGA, and TCGA databases) [77] to address whether overexpression of E2Fs and several mitotic regulators including BubR1, Hec1, Nek2, Mps1/TTK, and Sgo1 are correlated with survival outcomes. We found that overexpression of E2F1, E2F3, E2F1 and E2F3, BubR1, Hec1, Nek2, and Mps1/TTK and combined overexpression of all genes resulted in worse overall survival (Figure 3B). The same relationships were observed regarding relapse-free survival, except that underexpression of Sgo1 and overexpression of E2F1, E2F2, and E2F3 resulted in poor relapse-free survival (Figure 3C). Observations that E2F1, E2F2, and E2F3 correlate with relapse-free but not overall survival was confirmed by mining the METABRIC database, a database that encompasses over 2000 breast tumors, with cBioPortal (Figure 3D).

### Combined E2F overexpression results in slower growth of MCF10A cells and sustained expression and activity of mitotic regulators

**Figure 4:**
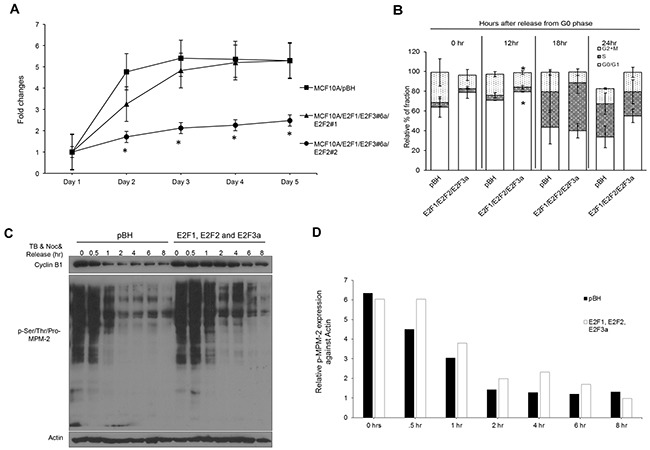
Combined E2F overexpression results in slower growth and a pause in G0/G1 in MCF10A cells **(A)** Cell proliferation/viability assay was performed using the CCK-8 kit. Data are presented as fold change relative to cell counts obtained at day 1. **(B)** Cells were serum-starved (0.2% serum) for 48 hours, released into media supplemented with 10% serum, and collected at 0, 12, 18, and 24 h after pulsing cells with 10 μM BrdU for 1 hour before harvesting. Cells were processed for FITC-BrdU/7-AAD staining, and specific cell cycle phases were presented as a percentage. **(C)** Cell protein lysates were prepared from cells treated with 2 mM thymidine for 18 hour, followed by nocodazole treatment (100 ng/mL) for 12 hour, washing off nocodazole and mitotic exit were measured by probing Western blots with cyclin B1 and pSer/Thr/Pro-MPM-2. **(D)** All bands from (C) were quantified with Image J.

To address whether combined E2F overexpression modifies the short-term growth of MCF10A cells, a proliferation/viability assay was performed (Figure 4A). Overexpression of E2F1, E2F2, and E2F3a resulted in slower growth and/or viability relative to cells expressing vector control, with population #2 displaying the slowest kinetics. The E2F activators were initially characterized by their ability to trigger proliferation in quiescent fibroblasts. To establish whether this was the case in mammary epithelial cells, MCF10A cells were cultured in a low concentration of serum (0.2%) for 48 hours and stimulated to enter the cell cycle by addition of 10% serum. Surprisingly, no differences in S phase fractions were detected at any time point after addition of serum (Figure 4B, Table 4). Only statistically significant increases in percentage of cells in G0/G1 and G2/M phases were found at the 12-hour time point in cells overexpressing E2F1, E2F2, and E2F3a, suggesting that the lower proliferation rate is due to a longer quiescent phase combined with less cells entering mitosis. Also, statistically significant differences were found in sub-G1 cells at the 0 hour time point. Overall, these results suggest that E2F overexpression in mammary epithelial cells resulted in minor changes in the cell cycle.

**Table 4:** Percentage of cells in each phase of the cell cycle

Hours after release	G0/G1 (%)	S (%)	G2+M (%)	Sub-G1 (%)
**0hr**				
pBABE-Hygro	64.23 ± 10.43	4.7 ± 3.92	30.57 ± 13.73	0.5 ± 0.32
E2F1/E2F2/E2F3a	79.030 ± 5.77	3.83±0.56	14 ± 5.59	3.13 ± 0.81*
**12hr**				
pBABE-Hygro	71.23 ± 0.5	5.23 ± 2.53	21.53 ± 2.63	2 ± 0.81
E2F1/E2F2/E2F3a	79.8 ± 0.78*	4.83 ± 1.73	14.73 ± 2.01*	0.67 ± 0.13
**18hr**				
pBABE-Hygro	43.37 ± 17.09	35.57 ± 20.67	19.33 ± 2.91	1.73 ± 0.8
E2F1/E2F2/E2F3a	40.13 ± 7.53	48.43 ± 10.63	10.87 ± 3.43	0.57 ± 0.23
**24hr**				
pBABE-Hygro	34.9 ± 11.57	34.4 ±10.8	15.87 ± 0.75	14.8 ± 13.92
E2F1/E2F2/E2F3a	54.97 ± 6.89	24.3 ±12.08	19.8 ± 4.94	0.97 ± 0.39

*p≤0.05

We have previously reported that silencing E2F3 affects mitosis and cytokinesis of Her2^+^ breast cancer cells [62, 63]. To determine whether E2F overexpression affects the mitotic machinery of MCF10A cells, we treated cells with thymidine (2 mM) for 18 hours to enrich cells in early S phase. Cells were allowed to continue S phase by releasing them into fresh media for 5 hours and then treating them with nocodazole (100 ng/mL) for 12 hours to enrich cells in M phase. This was followed by release from nocodazole into regular media to allow cells to continue mitosis and cytokinesis. While cyclin B, which is required for entry and exit of mitosis [20] was slowly downregulated over time in vector control cells, cyclin B levels were sustained much longer in cells overexpressing E2F1, E2F2, and E2F3a (Figure 4C). To address whether E2F overexpression affected the expression of other mitotic proteins, we probed lysates with phospho-Ser/Thr-Pro, MPM-2. The MPM-2 monoclonal antibody binds to a phospho-amino acid-containing epitope (peptides containing LTPLK and FTPLQ domains) present on more than 50 proteins of M-phase eukaryotic cells, thus representing a marker of mitosis [78]. E2F overexpression resulted in higher levels of phosphorylated proteins belonging to the MPM-2 complex throughout mitosis (Figure 4C and 4D). Consistent with statistically significant differences in the percentage of cells undergoing mitosis presented at the 12 hour time point in Figure 4B, the results presented here suggest that overexpression of the E2Fs leads to changes in important drivers of mitosis, including sustained cyclin B expression and higher phosphorylation levels of MPM-2.

### Combined E2F overexpression increases protein stability of mitotic regulators

**Figure 5:**
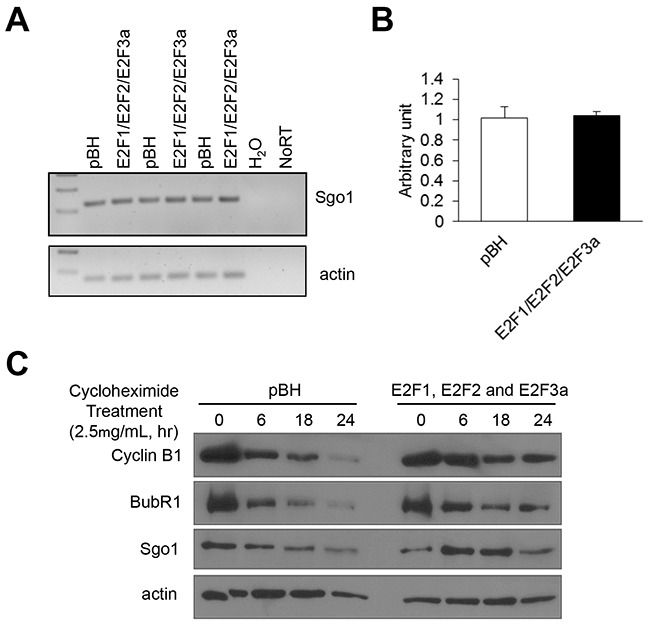
Combined E2F overexpression increases Sgo1 protein stability but not its transcription levels **(A)** Semi-quantitative PCR was performed for the Sgo1 transcript with cDNA synthesized from RNA extracted from cells expressing vector control (pBH) or cells overexpressing E2F1, E2F2, and E2F3a. Actin was used an internal control. **(B)** Real-time PCR was performed to quantify levels of Sgo1 mRNA. **(C)** Cells were treated with cycloheximide (2.5 μg/mL), cell protein lysates were prepared at the indicated time points, and Western blots were probed with antibodies recognizing cyclin B1, BubR1, and Sgo1. Actin was used as an internal control.

To identify molecular mechanisms by which overexpression of the E2Fs upregulates mitotic regulators, we first addressed whether combined E2F overexpression upregulates Sgo1 mRNA. To that end, total RNA was isolated from proliferating cells and semi-quantitative PCR and real-time PCR showed no differences in Sgo1 mRNA levels between vector control (pBH) and cells overexpressing E2F1, E2F2, and E2F3a (Figure 5A and 5B). Next, we explored whether combined E2F overexpression enhanced protein stability of Sgo1 and other mitotic regulators, including cyclin B1 and BubR1, since this mechanism has been invoked in the regulation of cyclin B by the E2Fs [59]. Cells were treated with cycloheximide (2.5 mg/mL) to stop new protein synthesis in order to measure protein stability, and cells were collected 0, 6, 18, and 24 hours after treatment. Although levels of these three mitotic regulators decreased over time in cells expressing vector control, protein levels of Sgo1, BubR1, and cyclin B were sustained for longer periods in cells overexpressing E2F1, E2F2, and E2F3a (Figure 5C). Overall, the results indicated that overexpression of E2Fs may stabilize cyclin B, BubR1, and Sgo1 proteins through direct or indirect mechanisms, either by increasing the stability of these mitotic regulators, or through regulating the transcription of factors that modulate degradation of these proteins.

### Transient Sgo1 knockdown in cells overexpressing three E2F activators induces centrosome amplification and chromosome instability

**Figure 6:**
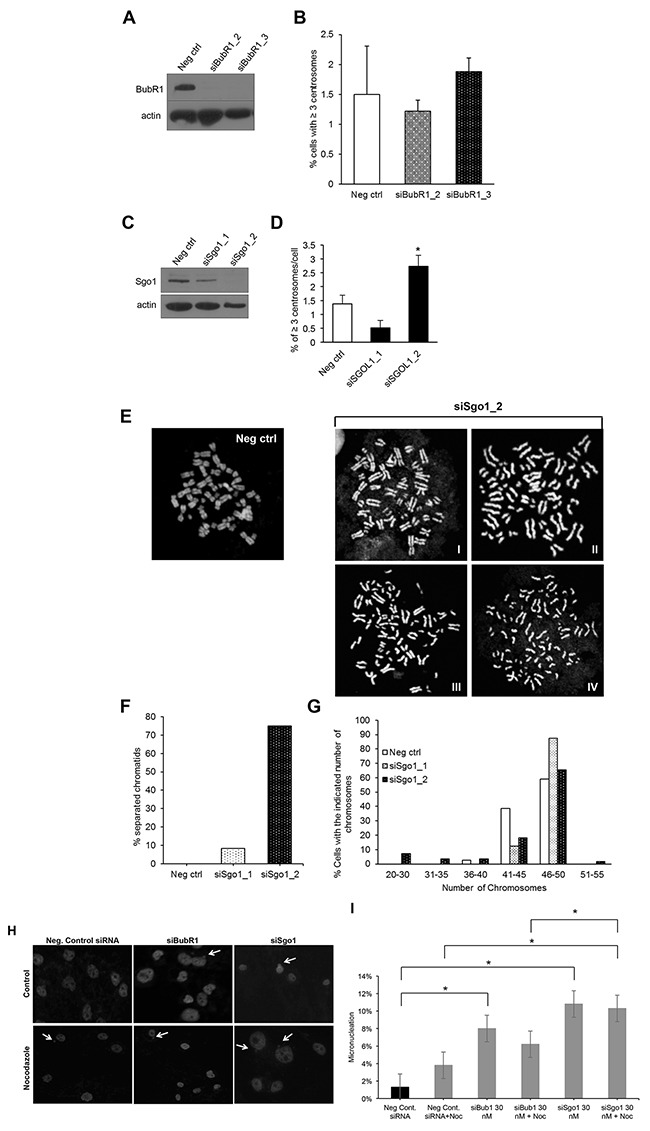
Transient knockdown of BubR1 or Sgo1 in cells overexpressing E2F1, E2F2, and E2F3a induces genomic instability, but only knockdown of Sgo1 induces centrosome amplification Cells overexpressing E2F1, E2F2, and E2F3a were transfected with siBubR1 or siSgo1 and their knockdown was confirmed by Western blot (**A**, and **C**, respectively), followed by the centrosome amplification assay (**B** and **D**, respectively). **(E)** Chromosome spreads were made from cells expressing E2F1, E2F2, and E2F3a transfected with control or siSgo1, and percentages of cells displaying premature separated chromatids **(F)**, or distribution of chromosome numbers **(G)** were quantified. The micronucleus assay was performed by immunofluorescence with DAPI in cells expressing control siRNA, siBubR1, or siSgo1 as measurement of chromosome instability **(H)**. **(I)** percentages of cells displaying micronuclei were calculated from three independent experiments (*P≤0.05).

Because we did not detect CA in cells overexpressing E2F1, E2F2, and E2F3a and since SAC regulators are highly upregulated in these cells, we addressed whether Sgo1 or BubR1 suppressed CA and CIN in this cell line. The Fukasawa group first described that upregulation of the BubR1 SAC regulator suppresses CA in p53-null cells, a phenomenon described as genomic convergence [79]. Genomic convergence is a mechanism by which cancer cells turn on a mitotic regulator that suppresses CA and CIN in order to increase fitness of cancer cells that have reached critically high levels of CA and CIN [80]. Thus, we speculated that, because BubR1 expression was high, it was acting as suppressor of CA. To test that hypothesis, we transiently knocked down BubR1; surprisingly, that silencing did not induce CA (Figure 6A and 6B). Next, we transfected cells with siRNA against Sgo1, one siRNA achieving partial silencing and one complete silencing (Figure 6C), and found that while its partial knockdown did not change percentages of CA, its complete reduction induced CA (Figure 6C and 6D). Shugoshin 1 (Sgo1), Japanese for “guardian spirit,” is a conserved kinetochore protein that protects centromere cohesion in fission yeast [17] and *Xenopus* [81]. Sgo1 protects phosphorylation of centromeric cohesin, which prevents premature chromatid separation [17, 19, 29, 81]. As expected, we detected various degrees of premature chromatid separation, from separated but closely intact sister chromatids (Figure 6E–6I and 6E-II) to totally separated sister chromatids (Figure 6E–6IV). Regardless of the degree of separation, we found about 70% of mitotic cells displaying separated chromosomes in mitosis (Figure 6F). We also calculated ploidy by counting chromosomes and found that complete knockdown of Sgo1 induced chromosome losses (Figure 6G). These data demonstrate that Sgo1 maintains genomic stability by regulating both chromosome cohesion and by preventing CA in cells overexpressing the E2F activators.

Recent work has shown that BubR1 helps recruit Sgo1 into centromeres and is part of a network that regulates premature SAC silencing [82]. To investigate whether Sgo1 and BubR1 play a role in the activation of the SAC in cells overexpressing the three E2Fs, we performed a micronucleus assay with siRNAs targeting BubR1 and Sgo1 as a measure for SAC dysfunction (Figure 6H and 6I). We found that the percentage of micronuclei, which is a measure of chromosome missegregation after cytokinesis and a measure of CIN [83, 84], was significantly higher in cells transiently transfected with siBubR1 (8.0%) and in cells with siSgo1 (10.8%) compared with the negative control (1.3%). We found significantly higher percentage of micronuclei in cells with 30 nM siSgo1 treated with nocodazole (100 ng/mL) for 18 hours (10.3%) than in the negative control cells treated with nocodazole (3.8%), as well as between siSgo1 treated with nocodazole (11%) versus siBubR1 cells treated with nocodazole (6.2%). However, we did not observe a significant percentage of micronuclei in cells with siBubR1 (8.0%) compared with siSgo1 (10.8%). Together, these data suggest that inactivation of BubR1 or Sgo1 similarly lead to inactivation of the SAC in asynchronously-growing cells [82], suggesting inactivation of Sgo1 leads to CA through other mechanisms.

### Transient Sgo1 knockdown decreases clonogenicity of cells by triggering apoptosis

**Figure 7:**
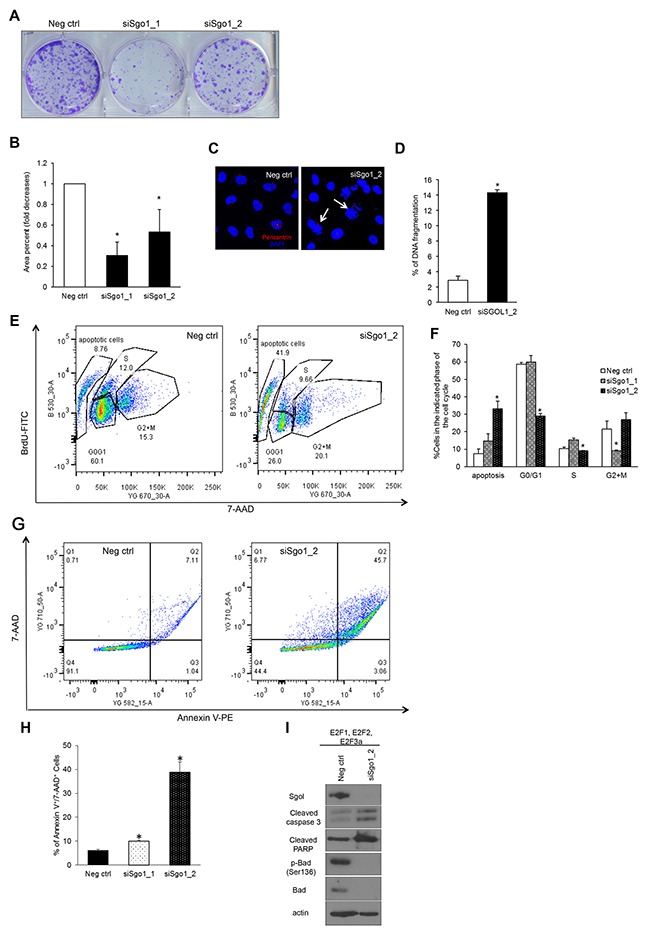
Transient Sgo1 knockdown decreases colony formation potential and induces apoptosis **(A)** Colony formation assay was done in MCF10A cells overexpressing E2F1, E2F2, and E2F3a transfected with control or siSgo1. **(B)** Colonies were quantified using Image J. **(C)** Nuclei were detected with DAPI, and the percentage of cells undergoing DNA fragmentation was calculated **(D)** (P≤0.05). **(E)** The BrdU/7-AAD assay was performed in MCF10A cells overexpressing E2F1, E2F2, and E2F3 transfected with control or siSgo1, and percentages of specific cell cycle phases were calculated **(F)**. **(G)** Annexin V staining was performed in MCF10A cells expressing E2F1, E2F2, and E2F3a with cells transfected with control or siSgo1, and percentages of Annexin V^+^/7-AAD^+^cells were calculated from three independent experiments **(H)**. **(I)** Western blots were performed with protein extracts from MCF10A cells expressing E2F1, E2F2, and E2F3a transfected with control or siSgo1 to measure levels of the indicated apoptotic markers.

To test whether Sgo1 downregulation in cells overexpressing the E2F activators affects cell viability, we performed a colony-forming assay and found that cells downregulated for siSgo1 displayed significantly decreased clonogenicity compared with negative control (Figure 7A and 7B). Decreased clonogenicity may be the result of cell cycle arrest or cell death. To address whether the decreased clonogenicity was due to cell death, we visualized nuclei by DAPI staining and found high levels of fragmented nuclei in cells completely silenced for Sgo1 (Figure 7C and 7D). To address whether cell death or pauses in cell cycle phases are responsible for decreased clonogenicity, we measured DNA content and cell cycle distribution using the BrdU/7-AAD assay (Figure 7E and 7F). Strikingly, we detected that partial silencing of Sgo1 resulted in a major reduction in cells undergoing G2/M relative to controls. On the other hand, complete depletion of Sgo1 by siRNA #2 led to approximately 30% of cells displaying a sub-G1 DNA content, indicative of the DNA fragmentation associated to cell death. We also found reduced percentages of cells in G0/G1 in cells transfected with siSgo1 clone #2. To confirm that the mechanism of cell death was apoptosis, we detected Annexin V by flow cytometry (Figure 7G and 7H), and indeed we detected about 10% cells positive for Annexin V using Sgo1 siRNA #1 (partial downregulation) and 40% with #2 (complete downregulation). To confirm that cells were undergoing apoptosis, we performed Western blot analyses with antibodies recognizing several apoptotic markers, including cleaved caspase 3, cleaved PARP, pBad (Ser136), and total Bad and observed changes on these proteins that are consistent with apoptosis [85–88] (Figure 7I).

To identify additional regulators of apoptosis in cells overexpressing E2Fs and silenced for Sgo1, we performed an antibody array that detected 247 total and phospho-specific sites from Full Moon Biosystems (Table 5). The top three upregulated proteins upon silencing of Sgo1 in cells that overexpress E2F1, E2F2, and E2F3 were Bcl-XL, p90RSK, and B-Raf, while the top downregulated genes were p53, HSP90-beta (Ser226), and SAPK/JNK (Thr183).

**Table 5:** Lists of potential Sgo1-mediated apoptosis targets

Genes Upregulated	Fold Change	Genes Downregulated	Fold Change
BCL-XL (Ab-47)	2.34	p53 (Ab-37)	0.72
P90RSK (Ab-359/363)	2.27	HSP90-beta (Ser226)	0.7
B-RAF (Ab-598)	2.19	SAPK/JNK (Thr183)	0.69
FADD (Ab-194)	1.99		
NFκB-p65 (Ser311)	1.95		
HSP90A (C-term)	1.76		
Fas (C-term)	1.76		
DAXX (Ab-668)	1.76		
Chk1 (Ab-286)	1.69		
Bax (N-term)	1.56		
Caspase 9 (Ab-196)	1.53		
ATRIP (Ab-68/72)	1.51		

## DISCUSSION

To mimic E2F deregulation in Her2^+^ cells, which display deregulation of three E2Fs [50, 62], we engineered MCF10A cells to overexpress E2F1, E2F2, and E2F3a at levels similar to those in Her2^+^ cells (Figure 1). The Western blots presented in Figure 2 indicate the ability of E2Fs to trigger G1/S-specific genes, with combined expression of the three E2F activators E2F1, E2F2, and E2F3 resulting in higher levels of cyclin D1, Cdk4, cyclin E, and p-Rb (Thr-821) relative to cells overexpressing individual E2Fs. Also, we observed increased levels of p53, p-p53 (Ser-15), and several CKIs, including p19^INK4D^, p21^CIP1^, and p27^Kip1^. Elevated levels of CKIs in cells expressing E2Fs are consistent with slow growth of MCF10A cells (Figure 4A) and the accumulation of cells in G0/G1 at 12 hours after serum release, suggestive of slower progression through G1 (Figure 4B). The high levels of G1/S regulators are consistent with the ability of E2Fs to regulate the G1/S transition [41, 89–91].

Because the E2Fs are known to control levels of genes that regulate the G1 and S phases, we focused on understanding how E2Fs control mitotic regulators that maintain genomic integrity, which is a novel activity. Although it is known that some mitotic regulators, including polo kinases, AURKA, Nek2, Cdk1, and cyclin B, are under the control of the E2Fs activators [56–58, 61, 62, 92–94], our present study is the first to demonstrate that overexpression of the E2F activators upregulates the expression of multiple proteins controlling various processes that affect mitosis, including proteins controlling centriole duplication (Mps1/TTK), chromosome attachment (Sgo1), and the SAC (Sgo1, Hec1, BubR1, and Mps1/TTK) (Figure 2). Importantly, the E2Fs and the mitotic regulators mentioned above are part of a much larger network of mitotic regulators that are deregulated in invasive breast cancers, including proteins that affect microtubule homeostasis, cell cycle control, the SAC and kinetochore structure and function, and centrosome regulation (Table 3). Given the high levels of SAC and mitotic regulators in cells overexpressing E2F1, E2F2, and E2F3a, we detected a significantly reduced fraction of G2/M cells 12 hours after serum addition to cells arrested in G0/G1 (Figure 4B, Table 4). We also found sustained expression of cyclin B and phosphorylation of MPM-2 in cells overexpressing E2Fs following a cell cycle block. Our results show two opposing signals impacting mitosis: one that triggers transit through M-phase (marked by increased levels of pMPM-2 and cyclin B) and a signal to delay exit caused by slower degradation of cyclin B (as modeled by the nocodazole treatments). These two opposing signals may explain the mild effect of combined E2F overexpression in G2/M, since overexpression led to decrease fractions of cells in G2/M only 12 hours after serum addition.

While upregulation of mitotic kinases *via* transcriptional control is a classical pathway by which E2Fs regulate these targets, indicated by the co-occurrence of E2F mRNAs and mRNAs of several mitotic kinases (Tables 2 and 3), we demonstrated that combined E2F activator overexpression upregulates several mitotic regulators, including cyclin B, Sgo1, and BubR1 (Figures 2 and 4) in part by enhancing protein stability (Figure 5C). As a result, it is plausible that overexpression of E2F activators deregulate the transcription of factors that modulate degradation of these proteins (for example, involved in the ubiquitination system), resulting in suppressed degradation of spindle assembly regulators, or that E2F proteins bind to degradation motifs of Sgo1, thus preventing its degradation.

Although we expected that combined E2F overexpression would induce CA and CIN higher than cells expressing single E2Fs, we observed similar levels of CA and CIN relative to cells expressing vector control. Because there is precedent that some mitotic regulators such as BubR1 suppress CA in p53-null cells, we hypothesized that either BubR1 or another mitotic regulator such as Sgo1 suppressed CA and CIN. The SAC is a mechanism that ensures normal chromosomal segregation, through the attachment of kinetochores to the spindle, before starting anaphase. Because we previously observed that single or combined expression of E2F increased the protein levels of regulators of the SAC including BubR1 (a suppressor of the APC/C activity) and Sgo1 (a suppressor of premature sister chromatids) (Figure 2), we performed transient knockdown of these genes (Figure 6 and Table 6). Silencing Sgo1 and BubR1 resulted in similar levels of micronuclei in asynchronously-cycling cells, suggesting that their inactivation equally led to failure of the SAC. However, when cells were treated with nocodazole, silencing Sgo1 resulted in a significantly higher number of micronuclei than cells silenced for BubR1, indicating that Sgo1 is a stronger regulator of the SAC under lack of tension. Nevertheless, we cannot rule out that lack of Sgo1 activity results in cytokinesis defects or centriole reduplication as possible mechanisms leading to CA. As of today, we do not know why Sgo1 suppressed CA in cells overexpressing the three E2Fs and not in cells expressing single E2Fs (as reported by us in [62]). Perhaps Sgo1 is cooperating with another suppressor turned on by co-expression of the E2Fs. Further global analysis, such as RNA seq or a solid state antibody array of cells co-overexpressing E2Fs *vs* cells overexpressing single E2Fs would be required to find out why the former did not induce CA and the later did.

**Table 6:** siRNA sequences

Genes	Sequences
siBubR1_2 F	5’-CUGAGGUUUUGAGAACUGCAAGGGGUC-3’
siBubR1_2 R	5’-GACUCCAAAACUCUUGACGUUCCCC-3’
siBubR1_3 F	5’-UUGACAUAUUACUCUCCUUCCCACCUU-3’
siBubR1_3 R	5’-AACUGUAUAAUGAGAGGAAGGGUGG-3’
siSgo1_1 F	5’-ACAGUAACCUUUCUCUUCAAAGATA-3’
siSgo1_1 R	5’-UAUCUUUGAAGAGAAAGGUUACUGUCU-3’
siSgo1_2 F	5’- CUGAAGACUUGUGAAAUCAAUGUTT-3’
siSgo1_2 R	5’-AAACAUUGAUUUCACAAGUCUUCAGGU-3’

**Figure 8:**
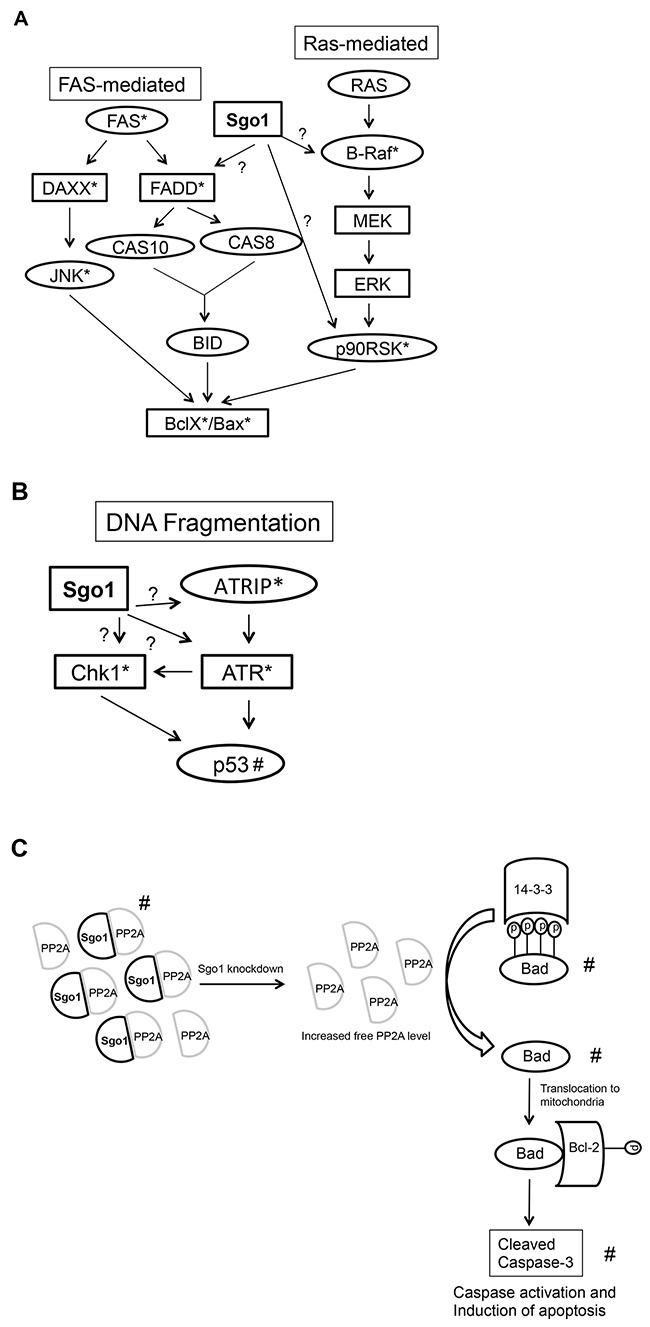
Apoptosis phosphor-Ab array generates potential targets of apoptosis signaled by silencing of Sgo1 in cells overexpressing E2Fs Proposed hypothetical apoptosis pathways that are mediated by Sgo1 **(A)** or DNA fragmentation pathway **(B)** based on apoptosis phospho Ab array. Proposed hypothetical apoptosis pathway based on our results **(C)**. *Detected by Ab array. #Detected by Western blot.

In mammary epithelial cells co-expressing the E2Fs, we found two mechanisms by which silencing Sgo1 decreases viability. First, partial silencing of Sgo1 resulted in a marked decrease in viability that correlates with decreases in cells undergoing G2/M and minor, but significant increases in percentages of cells undergoing apoptosis (Figure 7). Second, complete silencing of Sgo1 in MCF10A cells overexpressing the E2Fs induced apoptosis without G2/M arrest. We propose three models to explain the role of silenced Sgo1 in apoptosis (Figure 8). These models are based on Western blots (Figures 2 and 7) and a solid-state total and phospho-antibody array (Table 5), which identified upstream signaling pathways that may contribute to apoptosis, as well as apoptosis effectors that include cleaved PARP, cleaved caspase-3, and downregulated Bad. Model 1 proposes that silencing Sgo1 in E2F-overexpressing cells triggers apoptosis by activating the Fas- or B-Raf signaling pathways (Figure 8A). Another possibility is that the loss of cohesion and induction of CIN and/or deregulation of B-Raf pathway triggers checkpoint activation and cell death (Model 2). It proposes that silencing of Sgo1 in cells overexpressing E2Fs triggered checkpoint controls, resulting in the activation of ATR and increased phosphorylation of Chk1 (Figure 8B). In fact, the protein array detected potential checkpoint activation, as indicated by higher Chk-1 and ATRIP levels following knockdown of Sgo1 and ATRIP being an upstream activator of the Ataxia telangiectasia and Rad3-related protein (ATR) pathway [95], while Western blots detected higher p53 and p21 protein levels in cells expressing individual and/or combined E2Fs relative to MCF10A controls. This is consistent with E2F1's ability to activate p53 [96, 97].

Model 3 proposes that Sgo1 maintains high levels of Bad protein to suppress apoptosis and that silencing of Sgo1 resulted in low Bad levels and apoptosis (Figure 8C). PP2A is a major heterotrimeric serine/threonine phosphatase and is upregulated in cells overexpressing E2F1, E2F2, and E2F3 (Figure 2). The PP2A core is a dimer composed of the catalytic unit PP2AC and the 65-kDa anchoring A subunit A/PR65, and the third member of PP2A consists of regulatory subunits that are encoded by three multigene families [98], providing PP2A with versatile substrates and thereby involving it in various cellular processes including apoptosis. Silencing of Sgo1 may disrupt PP2A and Sgo1 complexes that are required to maintain centromere cohesion, resulting in more PP2A being available to dephosphorylate targets involved in apoptosis. There is experimental evidence to support that model; for example, phosphorylation of the cohesin subunit SA2 by Plk1 is critical for decreased chromosome cohesion [99] and Sgo1/PP2A complex dephosphorylates phospho-SA2, thereby protecting cohesin [100]. Bcl-2-associated death promoter protein (Bad) is a pro-apoptotic member of the Bcl-2 family, and dephosphorylation of Bad by either PP2A [101] or PP1 [102] translocates Bad into mitochondria where it interacts with Bcl-2 family members to trigger apoptotic cell death [103, 104]. More recently, our publication showed that the combination of silencing CDK4 and ionizing irradiation in triple negative breast cancer cells MDA-MB-231 and MDA-MB-468 lowered phospho-Bad-Ser136 levels and increased PP2A, which resulted in apoptosis [105]. Based on these observations, it is plausible that Sgo1-mediated apoptosis is mediated by the high levels of PP2A in cells overexpressing E2Fs. Finding the precise mechanisms of Sgo1-mediated apoptosis will give insights into how we can induce apoptosis in cancer models with SAC activation and how to develop small molecules targeting this pathway.

## MATERIALS AND METHODS

### Chemicals

Unless otherwise mentioned, all chemicals were purchased from Sigma-Aldrich (St. Louis, MO).

### Plasmids and cell lines

E2F1, E2F2, and E2F3a expressed from a pBABE-*hygro* backbone were a generous gift from Dr. Gustavo Leone (The Ohio State University). To overexpress two E2Fs, either E2F1 and E2F3a or E2F2 and E2F3a, E2F3a was cut from pBABE-*hygro* [106] vector and subcloned into pBABE-*puro*; then, MCF10A cells overexpressing either pBABE-*hygro*-E2F1 or -E2F2 were transfected with pBABE-*puro*-E2F3a using Trans*IT*-2020 transfection reagent (Mirus, Madison, WI). Cells underwent puromycin selection (2 μg/mL), and pools of clones were collected. To overexpress three E2Fs, E2F2 was cut from pBABE-hygro by *BamH*I/*EcoR*I digestion and subcloned into pcDNA3.1/3x myc-A vector. Cells overexpressing E2F1 and E2F3a were transfected with pcDNA3.1/3x myc-A-E2F2 using Trans*IT*-2020 transfection reagent, and cells underwent G418 selection. Pools of clones were collected. Cells overexpressing one, two, or three E2Fs were maintained in 50 μg/mL hygro, 50 μg/mL hygro and 2 μg/mL puro, 50 μg/mL hygro and 2 μg/mL puro, and 0.5 mg/mL G418 containing DMEM/F12 media (12500-096, GIBCO, Grand Island, NY) with 10% FBS, respectively. HCC1954, MDA-MB-231, and MDA-MB-468 cells were purchased from ATCC (Manassas, VA). HCC1954 cells were maintained in 10% FBS supplemented RPMI1640 (R8758, Sigma, St. Louis, MO), and MDA-MB-231 and MDA-MB-468 cells were grown in 10% FBS supplemented DMEM (11995-065, GIBCO). JIMT1 cells were kindly provided by Dr. Rita Nahta from Emory University and maintained in 10% FBS-supplemented DMEM.

### Cell cycle analysis and mitosis progression measurement

To analyze the cell cycle from serum-starved and released cells or siSgo1-transfected cells, we used the FITC-BrdU/7-AAD flow cytometry kit (57891, BD Pharmingen, San Jose, CA). Before harvesting, cells were pulse-labeled with 10 μM BrdU for 1 hour at 37°C. Cells were processed and immunostained according to the manufacturer's protocol, acquired in a BD LSRII flow cytometer, and analyzed with the Flowjo software (Ashland, OR). To observe changes in cyclin B and MPM-2 during mitosis, 2 × 10^6^ of vector control (pBABE-Hygro, pBH) and cells overexpressing the three E2Fs (E2F1, E2F2, and E2F3) were plated on a p100-mm plate, treated with 2 mM thymidine (T1895, Sigma, St. Louis, MO) for 18 hours, and released into fresh media without thymidine for 5-7 hours. Cells were then were treated with nocodazole (100 ng/mL, M1404, Sigma) for 12 hours, released into fresh media, and collected at 0, 0.5, 1, 2, 4, 6, and 8 hours. Proteins were extracted for Western blot analyses.

### Cell proliferation assay

One to two thousands cells were plated in 96-well plates in triplicates, and cell proliferation was measured for 5 days using the cell counting kit-8 (CCK-8) following the manufacturer's protocol (Dojindo Laboratories, Kumamoto, Japan).

### Real-time PCR

Total RNA was isolated using TRIzol following the manufacturer's protocol (Invitrogen, Grand Island, NY), and 2 μg of RNA was used to synthesize cDNA following the manufacturer's protocol (Promega, Madison, WI). We used 2 μL of 1:10 diluted cDNA for real-time PCR with iQ SYBR Green Supermix (170-8880, Bio-Rad, Hercules, CA). Actin was used as an internal control, and Sgo1 primer sequences have already been reported [68].

### Cycloheximide treatment

For cycloheximide (protein synthesis inhibitor) treatment, 1-2 × 10^6^ of pBH and three E2F overexpressing (E2F1/E2F2/E2F3a) cells were plated in p100-mm plates, and 2.5 or 5 μg/mL cycloheximide was added. Cells were collected 0, 6, 18, and 24 hours after treatment.

### siRNA transfection

We plated 3 × 10^5^ cells in p60 mm plates the day before transfection. Next, 15 μL of Lipofectamine RNAiMAX (13778075, Invitrogen) along with 200 pmol of Sgo1 siRNA constructs (Integrated DNA Technologies, Coralville, IA) [68], or BubR1 siRNA constructs (Table 6), or 5 μL of silencer negative control siRNA #1 (50 μM) (AM4611, Ambion, Grand Island, NY) were mixed in 300 μL of opti-MEM media, respectively, combined, and incubated for 25 minutes at room temperature. The mixtures were added to cells to transfect for either 24 or 48 hours.

### Colony-forming assay

Twenty-four hours after transfection, cells (2-3 × 10^3^ cells) were replated in 6-well culture plates. An average of 10 days after transfection, cells were fixed with 75% ethanol and stained with 1% crystal violet. Plates were scanned, and the images were processed with Image J to generate percentage of area.

### Immunofluorescence for centrosome amplification and DNA fragmentation assays

Cells were plated on a 4-well chamber slide and fixed in 4% paraformaldehyde for 10 minutes. After they were washed 3 times with 1× PBS for 5 minutes each, cells were permeabilized in 0.1% NP-40 for 10 minutes. Cells were washed and blocked in 10% normal goat serum (500622, Life Technologies) for 1 hour at room temperature, following overnight primary antibody incubation for pericentrin (ab4448, Abcam, Cambridge, MA). Alexa Fluor-conjugated antibodies (A11008, A11001, or A21069; Invitrogen) were used as secondary antibodies. For counterstaining, DAPI (4′,6′-diamidino-2-phenylindole) at 1 mg/ml was applied. Pictures were taken at ×40 magnification under a Zeiss Axioplan 2 fluorescence microscope. Two hundred cells were counted, percentage of cells with ≥ 3 centrosomes/cell was calculated to generate CA, and cells having extra DNA fragmentation were calculated to generate frequency.

### Chromosome spreads

Cells were plated in a p150-mm flask. When cells became confluent, they were treated with colcemid (final concentration of 100 ng/mL, #15210-040, GIBCO) for 2-4 hours at 37°C. Cells were collected by mitotic shake off and washed twice with HBSS (14175-095, GIBCO). Cells were then treated with hypotonic buffer (0.2% KCl, 0.2% sodium citrate, 10% FBS) for 20 to 30 minutes at 37°C, and the same amount of fixative (3 methanol:1 acetic acid) was added. After centrifugation, cells were washed twice with fixative and resuspended with it. Drops were made onto a clean slide coated with methanol, and a slide was stained with DAPI for microscopic visualization with Zeiss LSM-510 confocal microscope under ×100 magnification.

### Annexin V staining

Cells were prepared following manufacturer's protocol (BD 560930, BD Biosciences, San Jose, CA). Briefly, cells were washed twice with cold PBS and then re-suspended in 1× binding buffer(10 mM HEPES (pH 7.4), 0.14 M NaCl, 0.25 mM CaCl_2_) at a concentration of 1 × 10^6^ cells/mL. Then, 5 μL of Annexin V-PE and 5 μL of 7-AAD were added into 100 μL of cells, gently vortexed, and incubated for 15 minutes at room temperature in the dark. After 400 μL of 1× binding buffer was added into each tube, cells were acquired with BD LSRII flow cytometry and analyzed with flow J software.

### Apoptosis phospho-antibody array

Apoptosis phospho-antibody array was performed with E2F1/E2F2/E2F-3a overexpressing cells transiently transfected with either siSgo1_2 or silencer negative control. Cell lysates were prepared 30 hours after transfection, and slides coated with 247 site-specific and phospho-specific antibodies were hybridized with cell lysates according to the manufacturer's instructions (PAP247, Fullmoon Biosystems, Sunnyvale, CA). The slides were scanned with a microarray scanner, and signals were presented as fold changes by the manufacturer. In general, median signal intensity was extracted from array image for each spot on the array and the average signal intensity of replicate spots was determined. For normalization, median signal was determined from the median value of the Average Signal Intensity for all antibodies on the array (normalized data = average signal intensity of replicate spots / median signal). Finally, fold change between control and treatment was determined using the normalized data (fold change = treatment sample /control sample).

### Western blots

Cell protein lysates were prepared, and Western blotting was performed according to our published protocols [34, 105, 107]. The following primary antibodies were used in this experiment: E2F1 (3742, Cell Signaling), E2F2 (sc-633, Santa Cruz Biotechnology), E2F3 (sc-878, Santa Cruz Biotechnology), cyclin B1 (sc-245, Santa Cruz Biotechnology), Mad2 (ab70383, Abcam), Bcl2 (2870, Cell Signaling), pBcl2 (Ser70) (2827, Cell Signaling), pSer/Thr/Phe (9631, Cell Signaling), cleaved caspase 3 (9661, Cell Signaling), cleaved PARP (5625, Cell Signaling), Hec1 (GTX70268, GeneTex, Irvine, CA), pBad (Ser136) (4366, Cell Signaling), Bad (9268, Cell Signaling), Mps1/TTK (3255, Cell Signaling), PP2Ac (2259, Cell Signaling), and Sgo1 (ab58023, Abcam). β-actin antibody (4970, Cell Signaling) was used as a loading control. For secondary antibodies, either goat anti-rabbit HRP (sc-2004) or goat anti-mouse HRP (sc-2005, Santa Cruz Biotechnology) were used. Signals were detected by using a Lumigen TMA-6 reagent (Lumigen Inc, Southfield, MI). Image J software (NIH, Bethesda, MD) was used to quantify protein levels.

### Micronuclei assay

We plated 3 × 10^4^ cells overexpressing the three E2F (E2F1/E2F2/E2F3a) in 2-well chamber slides with 1 mL of cell media and incubated these overnight. Cells were transfected with silencer negative control siRNA #1 or 30 nM of siRNA constructs for BubR1 or Sgo1 and incubated for 48 hours. Next, 100 ng/mL nocodazole was added (or not) and allowed to incubate for 18 hours. Cells were fixed in 4% paraformaldehyde for 10 minutes. Cells were washed 3 times with 1× PBS for 5 minutes and permeabilized with 0.01% Triton-X 100/PBS for 10 minutes. Then, cells were washed with 1× PBS as described above prior to the staining with DAPI (1 μg/mL) for 5 minutes. Slides were allowed to seal overnight at room temperature. Pictures were taken at ×40 magnification using an Olympus BX60 fluorescence microscope. Two hundred cells were counted for each group; cells with micronucleus/total cells counted were calculated to obtain the percentage of micronucleation.

### Statistical analysis

Unless otherwise stated, Student *t* test was applied to compare the differences between vector control and three E2F overexpressing cell lines, with *P* value less than 0.05 considered significant. For cell proliferation assays, one-way ANOVA was used to compare the vector control and the two cell lines overexpressing of three E2F at each time point. For cell cycle progression analysis, SAS v9.3 (SAS Institute, Inc., Cary, NC) was used for analyses with a significant level of 0.05. A General Linear Regression model was used to obtain the least-squared mean (LSmean) of percentage of S, G0/G1, and G2+M at each cell line and time point.

## References

[1] Schvartzman JM, Sotillo R, Benezra R (2010). Mitotic chromosomal instability and cancer: mouse modelling of the human disease. Nature reviews Cancer.

[2] Perez de Castro I, de Carcer G, Malumbres M (2007). A census of mitotic cancer genes: new insights into tumor cell biology and cancer therapy. Carcinogenesis.

[3] Dominguez-Brauer C, Thu KL, Mason JM, Blaser H, Bray MR, Mak TW (2015). Targeting Mitosis in Cancer: Emerging Strategies. Mol Cell.

[4] Falchook GS, Bastida CC, Kurzrock R (2015). Aurora Kinase Inhibitors in Oncology Clinical Trials: Current State of the Progress. Semin Oncol.

[5] Wengner AM, Siemeister G, Koppitz M, Schulze V, Kosemund D, Klar U, Stoeckigt D, Neuhaus R, Lienau P, Bader B, Prechtl S, Raschke M, Frisk AL, von Ahsen O, Michels M, Kreft B (2016). Novel Mps1 Kinase Inhibitors with Potent Antitumor Activity. Mol Cancer Ther.

[6] Maachani UB, Kramp T, Hanson R, Zhao S, Celiku O, Shankavaram U, Colombo R, Caplen NJ, Camphausen K, Tandle A (2015). Targeting MPS1 Enhances Radiosensitization of Human Glioblastoma by Modulating DNA Repair Proteins. Mol Cancer Res.

[7] Tannous BA, Kerami M, Van der Stoop PM, Kwiatkowski N, Wang J, Zhou W, Kessler AF, Lewandrowski G, Hiddingh L, Sol N, Lagerweij T, Wedekind L, Niers JM, Barazas M, Nilsson RJ, Geerts D (2013). Effects of the selective MPS1 inhibitor MPS1-IN-3 on glioblastoma sensitivity to antimitotic drugs. J Natl Cancer Inst.

[8] Colombo R, Caldarelli M, Mennecozzi M, Giorgini ML, Sola F, Cappella P, Perrera C, Depaolini SR, Rusconi L, Cucchi U, Avanzi N, Bertrand JA, Bossi RT, Pesenti E, Galvani A, Isacchi A (2010). Targeting the mitotic checkpoint for cancer therapy with NMS-P715, an inhibitor of MPS1 kinase. Cancer Res.

[9] Huang LY, Lee YS, Huang JJ, Chang CC, Chang JM, Chuang SH, Kao KJ, Tsai YJ, Tsai PY, Liu CW, Lin HS, Lau JY (2014). Characterization of the biological activity of a potent small molecule Hec1 inhibitor TAI-1. Journal of experimental & clinical cancer research: CR.

[10] Hu CM, Zhu J, Guo XE, Chen W, Qiu XL, Ngo B, Chien R, Wang YV, Tsai CY, Wu G, Kim Y, Lopez R, Chamberlin AR, Lee EH, Lee WH (2015). Novel small molecules disrupting Hec1/Nek2 interaction ablate tumor progression by triggering Nek2 degradation through a death-trap mechanism. Oncogene.

[11] Wu G, Qiu XL, Zhou L, Zhu J, Chamberlin R, Lau J, Chen PL, Lee WH (2008). Small molecule targeting the Hec1/Nek2 mitotic pathway suppresses tumor cell growth in culture and in animal. Cancer Res.

[12] Clarke DJ, Gimenez-Abian JF (2000). Checkpoints controlling mitosis. BioEssays.

[13] Lew DJ, Burke DJ (2003). The spindle assembly and spindle position checkpoints. Annu Rev Genet.

[14] Malmanche N, Maia A, Sunkel CE (2006). The spindle assembly checkpoint: preventing chromosome mis-segregation during mitosis and meiosis. FEBS Lett.

[15] Fu G, Ding X, Yuan K, Aikhionbare F, Yao J, Cai X, Jiang K, Yao X (2007). Phosphorylation of human Sgo1 by NEK2A is essential for chromosome congression in mitosis. Cell Res.

[16] Fu G, Hua S, Ward T, Ding X, Yang Y, Guo Z, Yao X (2007). D-box is required for the degradation of human Shugoshin and chromosome alignment. Biochem Biophys Res Commun.

[17] Kitajima TS, Hauf S, Ohsugi M, Yamamoto T, Watanabe Y (2005). Human Bub1 defines the persistent cohesion site along the mitotic chromosome by affecting Shugoshin localization. Curr Biol.

[18] Liu H, Rankin S, Yu H (2013). Phosphorylation-enabled binding of SGO1-PP2A to cohesin protects sororin and centromeric cohesion during mitosis. Nat Cell Biol.

[19] McGuinness BE, Hirota T, Kudo NR, Peters JM, Nasmyth K (2005). Shugoshin prevents dissociation of cohesin from centromeres during mitosis in vertebrate cells. PLoS Biol.

[20] Pines J, Hunter T (1992). Cyclins A and B1 in the human cell cycle. Ciba Found Symp.

[21] Mardin BR, Schiebel E (2012). Breaking the ties that bind: new advances in centrosome biology. J Cell Biol.

[22] Harrison MK, Adon AM, Saavedra HI (2011). The G1 phase Cdks regulate the centrosome cycle and mediate oncogene-dependent centrosome amplification. Cell division.

[23] Fukasawa K (2011). Aberrant activation of cell cycle regulators, centrosome amplification, and mitotic defects. Horm Cancer.

[24] Sluder G, Khodjakov A (2010). Centriole duplication: analogue control in a digital age. Cell Biol Int.

[25] Nigg EA, Raff JW (2009). Centrioles, centrosomes, and cilia in health and disease. Cell.

[26] London N, Biggins S (2014). Signalling dynamics in the spindle checkpoint response. Nat Rev Mol Cell Biol.

[27] Tang Z, Shu H, Qi W, Mahmood NA, Mumby MC, Yu H (2006). PP2A is required for centromeric localization of Sgo1 and proper chromosome segregation. Dev Cell.

[28] Watanabe Y, Kitajima TS (2005). Shugoshin protects cohesin complexes at centromeres. Philos Trans R Soc Lond B Biol Sci.

[29] Tang Z, Sun Y, Harley SE, Zou H, Yu H (2004). Human Bub1 protects centromeric sister-chromatid cohesion through Shugoshin during mitosis. Proc Natl Acad Sci U S A.

[30] Lee NR, Kim HS, Kim YS, Kwon MH, Choi KS, Lee CW (2014). Regulation of the subcellular shuttling of Sgo1 between centromeres and chromosome arms by Aurora B-mediated phosphorylation. Biochem Biophys Res Commun.

[31] Tanno Y, Kitajima TS, Honda T, Ando Y, Ishiguro K, Watanabe Y (2010). Phosphorylation of mammalian Sgo2 by Aurora B recruits PP2A and MCAK to centromeres. Genes Dev.

[32] Yuan B, Xu Y, Woo JH, Wang Y, Bae YK, Yoon DS, Wersto RP, Tully E, Wilsbach K, Gabrielson E (2006). Increased expression of mitotic checkpoint genes in breast cancer cells with chromosomal instability. Clin Cancer Res.

[33] Marina M, Saavedra HI (2014). Nek2 and Plk4: prognostic markers, drivers of breast tumorigenesis and drug resistance. Front Biosci (Landmark Ed).

[34] Harrison Pitner MK, Saavedra HI (2013). Cdk4 and nek2 signal binucleation and centrosome amplification in a her2+ breast cancer model. PLoS One.

[35] Wang X, Zhou YX, Qiao W, Tominaga Y, Ouchi M, Ouchi T, Deng CX (2006). Overexpression of aurora kinase A in mouse mammary epithelium induces genetic instability preceding mammary tumor formation. Oncogene.

[36] Goepfert TM, Adigun YE, Zhong L, Gay J, Medina D, Brinkley WR (2002). Centrosome amplification and overexpression of aurora A are early events in rat mammary carcinogenesis. Cancer Res.

[37] Diaz-Rodriguez E, Sotillo R, Schvartzman JM, Benezra R (2008). Hec1 overexpression hyperactivates the mitotic checkpoint and induces tumor formation *in vivo*. Proc Natl Acad Sci U S A.

[38] Abbud RA, Takumi I, Barker EM, Ren SG, Chen DY, Wawrowsky K, Melmed S (2005). Early multipotential pituitary focal hyperplasia in the alpha-subunit of glycoprotein hormone-driven pituitary tumor-transforming gene transgenic mice. Mol Endocrinol.

[39] Sotillo R, Schvartzman JM, Socci ND, Benezra R (2010). Mad2-induced chromosome instability leads to lung tumour relapse after oncogene withdrawal. Nature.

[40] Sotillo R, Hernando E, Diaz-Rodriguez E, Teruya-Feldstein J, Cordon-Cardo C, Lowe SW, Benezra R (2007). Mad2 overexpression promotes aneuploidy and tumorigenesis in mice. Cancer Cell.

[41] Chen HZ, Tsai SY, Leone G (2009). Emerging roles of E2Fs in cancer: an exit from cell cycle control. Nature reviews Cancer.

[42] Lammens T, Li J, Leone G, De Veylder L (2009). Atypical E2Fs: new players in the E2F transcription factor family. Trends in cell biology.

[43] Cam H, Dynlacht BD (2003). Emerging roles for E2F: beyond the G1/S transition and DNA replication. Cancer Cell.

[44] Putzer BM (2007). E2F1 death pathways as targets for cancer therapy. J Cell Mol Med.

[45] Crosby ME, Almasan A (2004). Opposing roles of E2Fs in cell proliferation and death. Cancer Biol Ther.

[46] Johnson DG, Schneider-Broussard R (1998). Role of E2F in cell cycle control and cancer. Front Biosci.

[47] Paulson QX, McArthur MJ, Johnson DG (2006). E2F3a stimulates proliferation, p53-independent apoptosis and carcinogenesis in a transgenic mouse model. Cell Cycle.

[48] Pierce AM, Gimenez-Conti IB, Schneider-Broussard R, Martinez LA, Conti CJ, Johnson DG (1998). Increased E2F1 activity induces skin tumors in mice heterozygous and nullizygous for p53. Proc Natl Acad Sci U S A.

[49] Opavsky R, Tsai SY, Guimond M, Arora A, Opavska J, Becknell B, Kaufmann M, Walton NA, Stephens JA, Fernandez SA, Muthusamy N, Felsher DW, Porcu P, Caligiuri MA, Leone G (2007). Specific tumor suppressor function for E2F2 in Myc-induced T cell lymphomagenesis. Proc Natl Acad Sci U S A.

[50] Wu L, de Bruin A, Wang H, Simmons T, Cleghorn W, Goldenberg LE, Sites E, Sandy A, Trimboli A, Fernandez SA, Eng C, Shapiro C, Leone G (2015). Selective roles of E2Fs for ErbB2- and Myc-mediated mammary tumorigenesis. Oncogene.

[51] Pierce AM, Schneider-Broussard R, Gimenez-Conti IB, Russell JL, Conti CJ, Johnson DG (1999). E2F1 has both oncogenic and tumor-suppressive properties in a transgenic model. Mol Cell Biol.

[52] Muller H, Moroni MC, Vigo E, Petersen BO, Bartek J, Helin K (1997). Induction of S-phase entry by E2F transcription factors depends on their nuclear localization. Mol Cell Biol.

[53] Lukas J, Petersen BO, Holm K, Bartek J, Helin K (1996). Deregulated expression of E2F family members induces S-phase entry and overcomes p16INK4A-mediated growth suppression. Mol Cell Biol.

[54] Johnson DG, Schwarz JK, Cress WD, Nevins JR (1993). Expression of transcription factor E2F1 induces quiescent cells to enter S phase. Nature.

[55] Wu L, Timmers C, Maiti B, Saavedra HI, Sang L, Chong GT, Nuckolls F, Giangrande P, Wright FA, Field SJ, Greenberg ME, Orkin S, Nevins JR, Robinson ML, Leone G (2001). The E2F1, E2F2, and E2F3 Transcription Factors Are Essential for Cellular Proliferation. Nature.

[56] Ishida S, Huang E, Zuzan H, Spang R, Leone G, West M, Nevins JR (2001). Role for E2F in control of both DNA replication and mitotic functions as revealed from DNA microarray analysis. Mol Cell Biol.

[57] Ren B, Cam H, Takahashi Y, Volkert T, Terragni J, Young RA, Dynlacht BD (2002). E2F integrates cell cycle progression with DNA repair, replication, and G/M checkpoints. Genes Dev.

[58] Polager S, Kalma Y, Berkovich E, Ginsberg D (2002). E2Fs up-regulate expression of genes involved in DNA replication, DNA repair and mitosis. Oncogene.

[59] Lukas C, Sorensen CS, Kramer E, Santoni-Rugiu E, Lindeneg C, Peters JM, Bartek J, Lukas J (1999). Accumulation of cyclin B1 requires E2F and cyclin-A-dependent rearrangement of the anaphase-promoting complex. Nature.

[60] Peart MJ, Poyurovsky MV, Kass EM, Urist M, Verschuren EW, Summers MK, Jackson PK, Prives C (2010). APC/C(Cdc20) targets E2F1 for degradation in prometaphase. Cell Cycle.

[61] He L, Yang H, Ma Y, Pledger WJ, Cress WD, Cheng JQ (2008). Identification of Aurora-A as a direct target of E2F3 during G2/M cell cycle progression. J Biol Chem.

[62] Lee MY, Moreno CS, Saavedra HI (2014). The E2F activators signal and maintain centrosome amplification in breast cancer cells. Mol Cell Biol.

[63] Lee M, Oprea-Ilies G, Saavedra HI (2015). Silencing of E2F3 suppresses tumor growth of Her2+ breast cancer cells by restricting mitosis. Oncotarget.

[64] Fukasawa K (2007). Oncogenes and tumour suppressors take on centrosomes. Nat Rev Cancer.

[65] Godinho SA, Kwon M, Pellman D (2009). Centrosomes and cancer: how cancer cells divide with too many centrosomes. Cancer metastasis reviews.

[66] Anderhub SJ, Kramer A, Maier B (2012). Centrosome amplification in tumorigenesis. Cancer Lett.

[67] Chan JY (2011). A clinical overview of centrosome amplification in human cancers. Int J Biol Sci.

[68] Lee MY, Marina M, King JL, Saavedra HI (2014). Differential expression of centrosome regulators in Her2+ breast cancer cells versus non-tumorigenic MCF10A cells. Cell division.

[69] Neve RM, Chin K, Fridlyand J, Yeh J, Baehner FL, Fevr T, Clark L, Bayani N, Coppe JP, Tong F, Speed T, Spellman PT, DeVries S, Lapuk A, Wang NJ, Kuo WL (2006). A collection of breast cancer cell lines for the study of functionally distinct cancer subtypes. Cancer Cell.

[70] Zeng X, Shaikh FY, Harrison MK, Adon AM, Trimboli AJ, Carroll KA, Sharma N, Timmers C, Chodosh LA, Leone G, Saavedra HI (2010). The Ras oncogene signals centrosome amplification in mammary epithelial cells through cyclin D1/Cdk4 and Nek2. Oncogene.

[71] Debnath J, Muthuswamy SK, Brugge JS (2003). Morphogenesis and oncogenesis of MCF-10A mammary epithelial acini grown in three-dimensional basement membrane cultures. Methods.

[72] Rubin SM (2013). Deciphering the retinoblastoma protein phosphorylation code. Trends in biochemical sciences.

[73] Gao J, Aksoy BA, Dogrusoz U, Dresdner G, Gross B, Sumer SO, Sun Y, Jacobsen A, Sinha R, Larsson E, Cerami E, Sander C, Schultz N (2013). Integrative analysis of complex cancer genomics and clinical profiles using the cBioPortal. Science signaling.

[74] Cerami E, Gao J, Dogrusoz U, Gross BE, Sumer SO, Aksoy BA, Jacobsen A, Byrne CJ, Heuer ML, Larsson E, Antipin Y, Reva B, Goldberg AP, Sander C, Schultz N (2012). The cBio cancer genomics portal: an open platform for exploring multidimensional cancer genomics data. Cancer discovery.

[75] Ciriello G, Gatza ML, Beck AH, Wilkerson MD, Rhie SK, Pastore A, Zhang H, McLellan M, Yau C, Kandoth C, Bowlby R, Shen H, Hayat S, Fieldhouse R, Lester SC, Tse GM (2015). Comprehensive Molecular Portraits of Invasive Lobular Breast Cancer. Cell.

[76] Sorlie T, Perou CM, Tibshirani R, Aas T, Geisler S, Johnsen H, Hastie T, Eisen MB, van de Rijn M, Jeffrey SS, Thorsen T, Quist H, Matese JC, Brown PO, Botstein D, Eystein Lonning P (2001). Gene expression patterns of breast carcinomas distinguish tumor subclasses with clinical implications. Proc Natl Acad Sci U S A.

[77] Gyorffy B, Lanczky A, Eklund AC, Denkert C, Budczies J, Li Q, Szallasi Z (2010). An online survival analysis tool to rapidly assess the effect of 22,277 genes on breast cancer prognosis using microarray data of 1,809 patients. Breast Cancer Res Treat.

[78] Davis FM, Tsao TY, Fowler SK, Rao PN (1983). Monoclonal antibodies to mitotic cells. Proc Natl Acad Sci U S A.

[79] Oikawa T, Okuda M, Ma Z, Goorha R, Tsujimoto H, Inokuma H, Fukasawa K (2005). Transcriptional control of BubR1 by p53 and suppression of centrosome amplification by BubR1. Mol Cell Biol.

[80] Chiba S, Okuda M, Mussman JG, Fukasawa K (2000). Genomic convergence and suppression of centrosome hyperamplification in primary p53-/- cells in prolonged culture. Exp Cell Res.

[81] Salic A, Waters JC, Mitchison TJ (2004). Vertebrate shugoshin links sister centromere cohesion and kinetochore microtubule stability in mitosis. Cell.

[82] Jin F, Bokros M, Wang Y (2017). Premature Silencing of the Spindle Assembly Checkpoint Is Prevented by the Bub1-H2A-Sgo1-PP2A Axis in Saccharomyces cerevisiae. Genetics.

[83] Saavedra HI, Knauf JA, Shirokawa JM, Wang J, Ouyang B, Elisei R, Stambrook PJ, Fagin JA (2000). The RAS oncogene induces genomic instability in thyroid PCCL3 cells via the MAPK pathway. Oncogene.

[84] Saavedra HI, Fukasawa K, Conn CW, Stambrook PJ (1999). MAPK mediates RAS-induced chromosome instability. J Biol Chem.

[85] Jin Z, Gao F, Flagg T, Deng X (2004). Nicotine induces multi-site phosphorylation of Bad in association with suppression of apoptosis. J Biol Chem.

[86] Ruvolo PP, Deng X, May WS (2001). Phosphorylation of Bcl2 and regulation of apoptosis. Leukemia.

[87] Correia C, Lee SH, Meng XW, Vincelette ND, Knorr KL, Ding H, Nowakowski GS, Dai H, Kaufmann SH (2015). Emerging understanding of Bcl-2 biology: Implications for neoplastic progression and treatment. Biochim Biophys Acta.

[88] Youle RJ, Strasser A (2008). The BCL-2 protein family: opposing activities that mediate cell death. Nat Rev Mol Cell Biol.

[89] Trimarchi JM, Lees JA (2002). Sibling rivalry in the E2F family. Nature Reviews Molecular Cell Biology.

[90] Muller H, Helin K (2000). The E2F transcription factors: key regulators of cell proliferation. Biochim Biophys Acta.

[91] Herwig S, Strauss M (1997). The retinoblastoma protein: a master regulator of cell cycle, differentiation and apoptosis. Eur J Biochem.

[92] Tategu M, Nakagawa H, Sasaki K, Yamauchi R, Sekimachi S, Suita Y, Watanabe N, Yoshid K (2008). Transcriptional regulation of human polo-like kinases and early mitotic inhibitor. J Genet Genomics.

[93] Black EP, Hallstrom T, Dressman HK, West M, Nevins JR (2005). Distinctions in the specificity of E2F function revealed by gene expression signatures. Proc Natl Acad Sci U S A.

[94] Russo AJ, Magro PG, Hu Z, Li WW, Peters R, Mandola J, Banerjee D, Bertino JR (2006). E2F-1 overexpression in U2OS cells increases cyclin B1 levels and cdc2 kinase activity and sensitizes cells to antimitotic agents. Cancer Res.

[95] Cortez D, Guntuku S, Qin J, Elledge SJ (2001). ATR and ATRIP: partners in checkpoint signaling. Science.

[96] Brown DI, Lassegue B, Lee M, Zafari R, Long JS, Saavedra HI, Griendling KK (2014). Poldip2 knockout results in perinatal lethality, reduced cellular growth and increased autophagy of mouse embryonic fibroblasts. PLoS One.

[97] Qin XQ, Livingston DM, Kaelin WG, Adams PD (1994). Deregulated transcription factor E2F-1 expression leads to S-phase entry and p53-mediated apoptosis. Proc Natl Acad Sci U S A.

[98] Janssens V, Goris J (2001). Protein phosphatase 2A: a highly regulated family of serine/threonine phosphatases implicated in cell growth and signalling. Biochem J.

[99] Hauf S, Roitinger E, Koch B, Dittrich CM, Mechtler K, Peters JM (2005). Dissociation of cohesin from chromosome arms and loss of arm cohesion during early mitosis depends on phosphorylation of SA2. PLoS Biol.

[100] Kitajima TS, Sakuno T, Ishiguro K, Iemura S, Natsume T, Kawashima SA, Watanabe Y (2006). Shugoshin collaborates with protein phosphatase 2A to protect cohesin. Nature.

[101] Chiang CW, Harris G, Ellig C, Masters SC, Subramanian R, Shenolikar S, Wadzinski BE, Yang E (2001). Protein phosphatase 2A activates the proapoptotic function of BAD in interleukin- 3-dependent lymphoid cells by a mechanism requiring 14-3-3 dissociation. Blood.

[102] Ayllon V, Cayla X, Garcia A, Roncal F, Fernandez R, Albar JP, Martinez C, Rebollo A (2001). Bcl-2 targets protein phosphatase 1 alpha to Bad. Journal of immunology.

[103] Tzivion G, Avruch J (2002). 14-3-3 proteins: active cofactors in cellular regulation by serine/threonine phosphorylation. J Biol Chem.

[104] Datta SR, Katsov A, Hu L, Petros A, Fesik SW, Yaffe MB, Greenberg ME (2000). 14-3-3 proteins and survival kinases cooperate to inactivate BAD by BH3 domain phosphorylation. Mol Cell.

[105] Hagen KR, Zeng X, Lee MY, Tucker Kahn S, Harrison Pitner MK, Zaky SS, Liu Y, RM OR, Deng X, Saavedra HI (2013). Silencing CDK4 radiosensitizes breast cancer cells by promoting apoptosis. Cell division.

[106] Morgenstern JP, Land H (1990). Advanced mammalian gene transfer: high titre retroviral vectors with multiple drug selection markers and a complementary helper-free packaging cell line. Nucleic Acids Res.

[107] Adon AM, Zeng X, Harrison MK, Sannem S, Kiyokawa H, Kaldis P, Saavedra HI (2010). Cdk2 and Cdk4 regulate the centrosome cycle and are critical mediators of centrosome amplification in p53-null cells. Mol Cell Biol.

